# Head to Head Comparison between Different 3-Dimensional Echocardiographic Rendering Tools in the Imaging of Percutaneous Edge-to-Edge Mitral Valve Repair

**DOI:** 10.3390/jcdd8070073

**Published:** 2021-06-24

**Authors:** Gloria Tamborini, Valentina Mantegazza, Anna Garlaschè, Manuela Muratori, Laura Fusini, Sarah Ghulam Ali, Claudia Cefalù, Gianpiero Italiano, Paola Gripari, Anna Maltagliati, Marco Penso, Mauro Pepi

**Affiliations:** Centro Cardiologico Monzino, IRCCS, 20138 Milan, Italy; Valentina.Mantegazza@ccfm.it (V.M.); anna.garlasche@ccfm.it (A.G.); Manuela.Muratori@ccfm.it (M.M.); laura.fusini@ccfm.it (L.F.); Sarah.Ghulamali@ccfm.it (S.G.A.); claudia.cefalu@ccfm.it (C.C.); gianpiero.italiano@ccfm.it (G.I.); Paola.Gripari@ccfm.it (P.G.); anna.maltagliati@ccfm.it (A.M.); Marco.Penso@ccfm.it (M.P.); mauro.pepi@ccfm.it (M.P.)

**Keywords:** three-dimensional transesophageal echocardiography, transesophageal echocardiographic monitoring, mitral valve prolapse, mitraClip procedure

## Abstract

MitraClip (MC) is the most common percutaneous treatment for severe mitral regurgitation (MR). An accurate two-dimensional and three-dimensional echocardiographic (3DTEE) imaging is mandatory for the optimal procedural result. Recently transillumination 3DTEE rendering (3DTr) has been introduced integrating a virtual light source into the dataset and with the addition of glass effect (3DGl) allows to adjust tissue transparency improving depth perception and anatomical structure delineation in comparison with the standard 3DTEE (3DSt). The aim of this retrospective study in 30 patients undergoing MC, was to compare 3DSt, 3DTr, and 3DGl in mitral valve (MV) evaluation and procedural result assessment. 3DTEE acquisitions obtained before and after MC were processed with 3DSt, 3DTr, and 3DGl rendering. Each reconstruction was scored for quality and for ability to recognize MV anatomy, MR origin, clip position, dimension and grasping. Imaging quality was judged good or optimal in 52%, 76%, and 96% in 3DSt, 3DTr, and 3DGl reconstructions respectively. In 26/30 patients a diagnostic incremental value was found with 3DTr vs. 3DSt and in 15/26 with 3DGl vs. 3DTr and 3DSt. Only 3DGl with perpendicular cropping of the clip allowed to visualize and measure the grasped portion of each mitral leaflets. 3DTEE imaging during MC may be improved by 3DTr and 3DGl providing a better evaluation of MV, of leaflet grasping and of residual MR jets after MC.

## 1. Introduction

MitraClip (MC) system is the most commonly implanted transcatheter technology for the management of severe mitral regurgitation (MR) [1,2,3]. Patients from the initial experience (mainly trials) had to fulfill strict echocardiographic criteria to be considered suitable for MC, which largely limited its indications [4,5]. Nowadays, the large accumulated experience in parallel with continuous technical advancements broadened the indications of the procedure in both primary (degenerative) or secondary (functional) MR. Therefore, diagnosis of mitral valve (MV) pathology and monitoring of the procedure becomes more complex and selection of candidates, planning, and guiding of the procedure requires a very detailed support by 2-dimensional (2D) and 3-dimensional (3D) transesophageal echocardiography (TEE) [6,7,8].

Recently a new 3D rendering tool known as transillumination (3DTr), which integrates a virtual light source into the data set, and transparency or “glass” effect (3DGl) that allow the operator to adjust the degree of cardiac and extracardiac structure transparency further improved visualization and delineation of anatomy and pathology and improved localization of regurgitant jets compared with the standard 3D rendering (3DSt) [9,10,11,12]. It has been hypothesized in very preliminary reports that the addition of this new tissue 3DTr tool and its integration with 3D color Doppler would result in improved anatomic detail, border definition and localization of pathology, including regurgitant jet origin, compared with 3DSt rendering both with and without 3DTr [13,14].

The aims of this study in patients undergoing MitraClip were to compare 3DSt rendering, 3DTr and 3DGl in 3 settings: (a) mitral valvular morphology (b) color jet origin (c) assessment of final procedure results (optimal leaflets capture and residual MR jets origin).

## 2. Materials and Methods

The study population included 30 consecutive patients who underwent MV repair with the MitraClip system (Abbott Lake Bluff, IL) at Centro Cardiologico Monzino IRCCS between November 2019 and November 2020.

All patients were affected by severe MR (secondary: 5 cases or primary: 25 cases). MR severity was determined by integrating 6 criteria including qualitative (Color Doppler jet characteristics, pulmonary vein flow pattern) and quantitative (vena contracta width, regurgitant volume, regurgitant fraction, and effective regurgitant orifice area) measurements [15,16,17].

Cases with failure of the procedure (aborted procedure) were not observed in this consecutive series.

The local research committee approved this retrospective study protocol and all study participants provided written informed consent.

A dedicated “heart valve team” of cardiologists, cardiovascular surgeons, and anesthesiologists referred the patients for MitraClip procedure on the basis of current guidelines [1,2], MV anatomy, and the presence of high-risk criteria (including logistic European System for Cardiac Operative Risk Evaluation score > 20%) or other comorbidities, such as neurologic disorders and respiratory diseases.

Clinical exclusion criteria included recent myocardial infarction, any interventional or surgical procedure within one month, renal insufficiency, active infections, history of rheumatic heart disease, and prior MV surgery. The pre-procedural imaging protocol included complete 2D and 3D transthoracic examinations (EPIQ 7C system, X5-1 probe, Philips Medical Systems, Andover, MA, USA) performed the day before MitraClip. Transcatheter MV repair was performed under general anesthesia and guided by fluoroscopy and 2DTEE and 3DTEE (EPIQ systems, X7-2T probe, Philips Medical Systems, Andover, MA, USA).

All steps of the procedure were carefully monitored by 2DTEE and 3DTEE: pre-procedural, trans-septal puncture, left atrial positioning of exchange guide wire and guide catheter, advancement of the delivery system, alignment of the arms perpendicular to the line of coaptation, capture of the leaflets, adequacy of capture with adequate achievement of a double orifice, release of the clip.

Digital acquisitions of 3DTEE images throughout the procedure were performed including 3D full volume, zoom real time, and 3D color of the region of interest.

Two expert cardiologists (blinded to each other) retrospectively analyzed 3DTEE acquisitions (EPIQ system, Philips) obtained during the intraprocedural TEE before and after clip implantation. Zoomed and full volume acquisitions with and without color Doppler signal and focused on MV apparatus, were independently processed by the 2 operators with 3DSt, 3DTr and 3DGl rendering. These new methods allow instantaneous changes of rendering obtained with changes of real time or acquired images by touching the screen, moving the light position (3DTr) and applying transparency (3DGl) [13]. Operators tried to obtain the ideal rendering of each clip by adjusting gains, compression, viewing prospective and cropping the data set, when useful.

In all the patients, each reconstruction was scored using a Likert scale from 1 to 5 (1 the worst to 5 the best) [9] for quality and for the ability to recognize mitral anatomy, to identify the regurgitant jet origin, to evaluate clip position and insertion after clip implantation.

**Figure 1:**
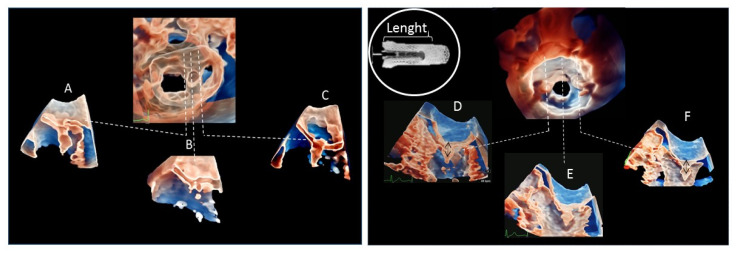
Left Panel: three-dimensional transesophageal (3DTEE) surgical view of a single MitraClip (MC) implantation with a typical optimal final result. From (**A**–**C**) consecutive longitudinal 3DTEE sections with transparency rendering from anterolateral to posteromedial. (**A**): visualization of the MC and the grasped anterior leaflet. (**B**: both leaflets are well visualized and the inserted leaflet portion may be measured. (**C**): MC depth is well defined. Right panel: 3DTEE reconstruction with transparency rendering of the mitral valve from left ventricular view in an atypical case with the insertion of 2 MC resulting in a single central orifice. From (**D**–**F**) consecutive longitudinal sections from anterolateral to posteromedial. (**D**,**F**) longitudinal sections of the 2 MC implanted in lateral (**D**) and medial (**F**) position, respectively. (**E**): longitudinal section of the central residual mitral orifice. White encircle panel: MC length that has been compared to the 3D images. Black arrows showing the MC length measurements corresponding with the nominal dimensions.

From the volumetric data sets, specific cut planes were used to obtain longitudinal section of each implanted clip from a medio-lateral view adapted to clip position and MV anatomy to evaluate objectively these new methods, measurements of clip arm length were obtained and compared to nominal measurements in either smaller (NTR) or larger (XTR) models. Moreover, operators utilized 3DGl to obtain cross clip sections of the grasped portion of each mitral leaflet and annotated the visualized portion of the leaflet and measured its length (Figure 1).

Continuous variables are presented as mean ± standard deviation or media (25th–75th percentile) as appropriate. A one-way analysis of variance was used to compare the Likert scores for the three different display modality. Results are expressed as mean ± SD. Significance of differences (*p* < 0.05) was analyzed by the paired Student’s *t*-test for the difference between mean values. In 15 randomly selected patients, 3DTEE measurements were independently calculated by 2 examiners and repeated by another observer. Inter- and intra-observer variabilities in clip length measurements are reported in terms of intraclass correlation coefficients (ICCs).

## 3. Results

All 30 patients included in the study underwent successful MC implantation. Mean MR severity decreased from 3.8 ± 0.7 to 1.5 ± 0.7 being post-procedural MR ≤ 2 in 28/30 cases. In 2 patients residual MR was moderate and moderate to severe, respectively. Specifically, in one case with functional MR (insertion of a small size clip) the mean mitral diastolic gradient was 6 mmHg and although the presence of moderate (3+) residual MR no more clips were implanted. In the second patient with degenerative MR, 2 large size clips were inserted and central regurgitation was reduced. However, after the second clip delivery a breach cleft was observed associated with a moderate to severe MR. Number and type of the implanted clips are reported in Table 1.

**Figure 2:**
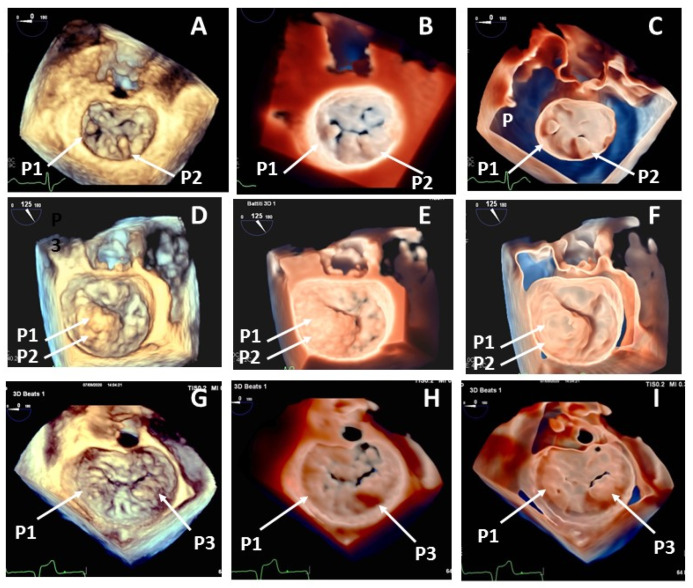
Head-to-head comparison among standard 3DTEE rendering (left panels-**A**,**D**,**G**), transillumination (central panels-**B**,**E**,**H**) and transparency (right panels-**C**,**F**,**I**) in 3 cases with mitral valve prolapse visualized in the surgical view. Case 1 (Upper panels): Segmented-loculated P1 and bilobed P2 prolapses. Case 2 (mid panels): Large P2 prolapse associated with a P1 prolapse. Case 3 (Bottom panels): P1 and P3 Prolapses. Shadowing and transparency improve delineation of scallop borders and malcoaptation areas.

Quality of 3DSt reconstructions was sufficient in all cases and good or optimal in 52%. 3DTr and 3DGl reconstructions were judged as good or optimal, respectively, in 76% and 94% of cases improving the quality of 3DSt imaging (Figure 2).

In 26 out of the 30 cases a diagnostic incremental value, in terms of improvement in delineation of scallop, cleft, chordae and clips, was found with 3DTr vs. 3DSt and in 15 cases with 3DGl vs. 3DTr and 3DSt. Table 2 shows quality score comparison among the 3 different 3DTEE display modalities concerning the anatomical features definition.

### 3.1. Mitral Valve Anatomy 

**Figure 3:**
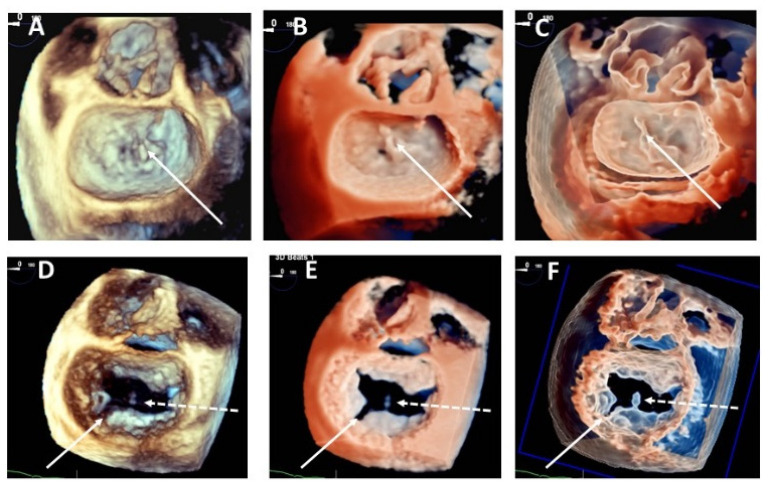
Head-to-head comparison among standard 3DTEE surgical view rendering (left panels-**A**,**D**), transillumination (central panels) and transparency (right panels) in 2 cases with chordal rupture and clefts. Upper panels: 3DTr (**B**) and 3DGl (**C**) rendering significantly improve chordal rupture visualization (arrows) in a flail P2. Lower panels: 3DTr (**E**) and 3DGl (**F**) rendering significantly improve cleft visualization between P2 and P1 (arrows) TGl allows a more precise delineation of a P2 flail and chordal rupture (dotted arrows).

Additional value in mitral anatomy evaluation with 3DTr was found in 9 patients, the addition of 3DGl modality improved diagnostic definition in other 8 cases. Involved prolapsing scallop morphology (5 cases), chordal rupture definition (8 cases), cleft identification (4 cases) were the principal point of improvement with the new techniques (Figure 3).

### 3.2. Regurgitant Color Jets

**Figure 4:**
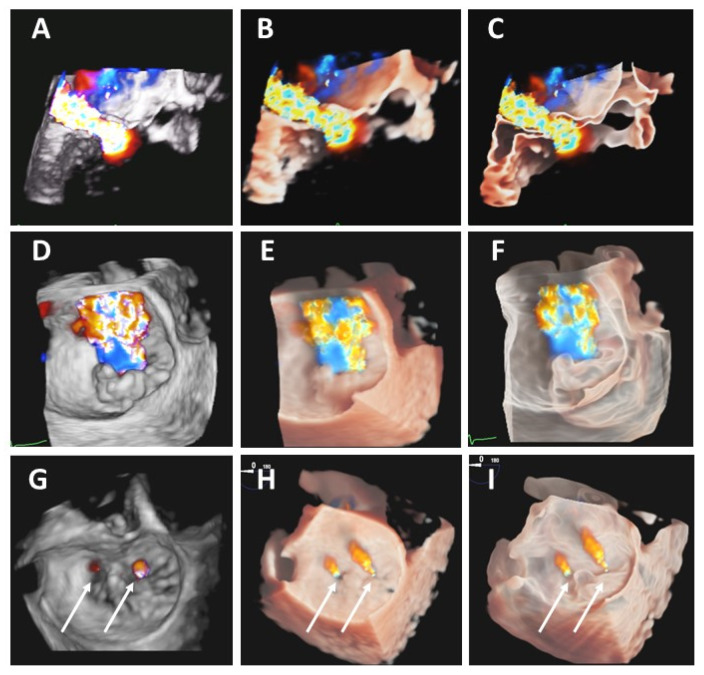
Head-to-head comparisons between standard 3DTEE (3DTs, left panels), transillumination (3DTr, central panels) and glass feature (3DGl, right panes) merged with color Doppler rendering in a case of mitral valve regurgitation due to P2 prolapse before and after a Mitra-clip implantation. In panels (**A**–**C**) longitudinal view of color jet and flow acceleration shape for calculation of effective regurgitant orifice area using 3D proximal isovelocity surface area. In panels (**D**–**F**), 3DTr and 3DGl improve delineation of the borders of the MV regurgitant orifice in the surgical view of the valve. In the lower panels (**G**–**I**) post-procedural reconstructions after the implantation of a central clip demonstrating the more defined identification of 2 residual color jets with transillumination and transparency modalities (arrows).

In all the patients color jet origin was better defined with 3DTr and 3DGl, and in 8 cases only 3DTr and 3TGl allowed a precise definition of regurgitant jet origin. In 3 patients a clear intra-clip color jet was identified (Figure 4).

### 3.3. Clip Implantation

**Figure 5:**
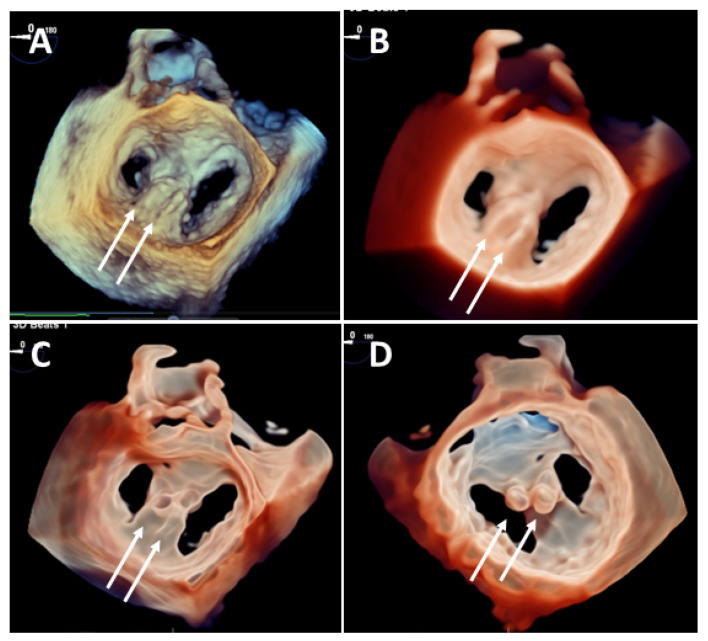
Comparisons of the standard 3DTEE (**A**), transillumination (**B**) and transparency (**C**,**D**) in the evaluation of 2 clips implanted side by side in the A2-P2 region (arrows). The perspective in (**A**–**C**) is the surgical view while in (**D**) is the ventricular view. Transillumination with addition of glass rendering allows an excellent definition of the 2 orifices and the more precise identification of clip location.

In all the patients 3DTEE of the MV was acquired and reconstructed after deployment. Forty-three clips were implanted and visualized in the 30 patients (Figure 5).

**Figure 6:**
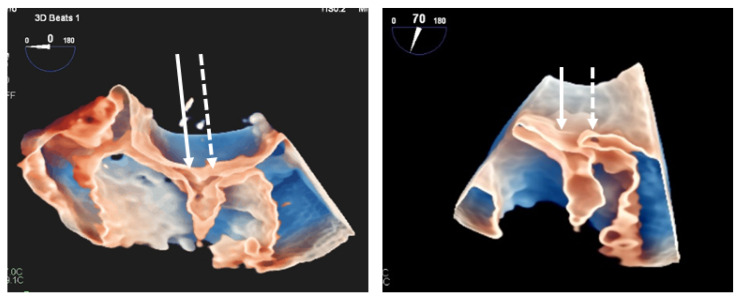
Two examples of grasped leaflet visualization with 3DTEE in transillumination plus transparency modality. Left panel: both anterior (arrow) and posterior (dotted arrow) leaflet are perfectly grasped by the clip in this patient. Right panel: In this case the anterior leaflet is well grasped while the posterior leaflet (dotted arrow) has not been captured.

3DTr and 3DGl allowed a significant improvement in clip visual evaluation and analysis of leaflet grasping modality. As concerns the quantitative analysis in 2 cases with commissural clip implantation despite clips were correctly visualized, measurements were not feasible due to suboptimal cropping and alignment in these regions. Therefore, quantitative analysis was performed in 41 out of 43 clips (95.4%), showing a good correlation between 3DTEE and nominal device dimensions. Indeed, the average length difference between the nominal (NTR 9 mm, XTR 12 mm) and measured (NTR 9.3 ± 0.4 mm, XTR 11.9 ± 0.6 mm) values was 0.35 ± 0.4 for NTR and 0.02 ± 0.6 for XTR respectively. Intra-observer variability of length as interclass correlation coefficients were 0.951, while inter-observer variability was 0.923. Only the addition of tissue transparency (3DGl) together with perpendicular cropping of the clip allowed to visualize the grasped portion of mitral leaflets (Figure 6).

**Figure 7:**
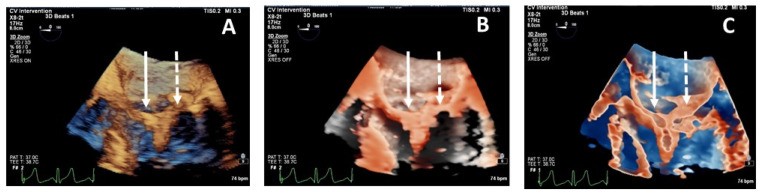
Head-to-head comparison among standard 3DTEE rendering panel (**A**), transillumination panel (**B**) and transparency panel (**C**) in clip grasping evaluation. Only transparency rendering allows one to visualize that anterior leaflet is well grasped (arrow) while the posterior leaflet (dotted arrow) has not been captured correctly and it appears short and folded into the arms of the clip.

Interestingly, in one patient, the grasped portion of the MV posterior leaflet appeared particularly short (<5 mm) and folded. In this patient a partial detachment of the clip was observed at 6 month follow up (Figure 7).

## 4. Discussion

The main findings of this study are three-fold: (a) new 3DTEE rendering tools may further improve the evaluation of pre-procedural MV anatomy and MC monitoring; (b) 3DTr and 3DGl further improve a qualitative imaging score throughout the procedure; (c) grasping of the leaflets, position and measurements of the device and precise definition of size and origin of the residual MR are the main additional clinical values of these new rendering tools. The MC procedure is performed under fluoroscopic and TEE guidance and these images are simultaneously displayed on a screen. Effective communication between the echocardiographer and the interventionalist is paramount to facilitate procedural guidance and optimize outcomes. This method may facilitate orientation and catheter manipulation by the interventionalist and it is therefore obvious that quality of echo imaging is essential [7,18,19]. In MV disease, 3D imaging enhances the evaluation of all anatomic and functional details, including subcomponents of the MV apparatus (annulus, leaflets, chordae and papillary muscles) [20,21]. Both qualitative and quantitative evaluations of functional and degenerative MV disease have been substantially improved by 3D echocardiography [18,19,20,21,22]. Recently 3DTTE and 3DTEE diagnostic accuracy has been further improved thanks to new transducers, software and transillumination techniques [7,9,10,11,12,13,14]. Transillumination is user friendly: a touch screen allows operator to change the light source orientation, creating a 3D reconstruction that markedly emphasize a shadow effect. Thus, the most relevant innovation is the perception of depth, which is notably improved with its use. In association with transillumination the new transparency modality may help to clearly delineate pathologic areas such as prolapsed valve leaflet scallops empowering a clear distinction of cardiac and extra-cardiac structures. Karagodin et al. [9] demonstrated that experts perceived an incremental value of the transparency mode compared with transillumination without transparency and standard 3D rendering in terms of ability to recognize anatomy, identify pathology, depth perception and border delineation. In our study 2 experts compared 3DSt, 3DTr and 3DGl by evaluating several data concerning MC procedure. Quality of images clearly improves with these 2 new tools, and imaging of clips details as well as grasping of the leaflets took advantages by the transparency effect. Since the majority of previous and our observations are subjective and qualitative, we also compared the nominal size of the clip to 3DGl data showing a very good correspondence between arm length measured by 3D and the implanted device. These 3DGl measurements were independent of the models (NTR and XTR that have different dimensions) and confirm that this new 3D glass or transparency effect allows a true imaging of intracardiac structures, permitting quantitative measurement not feasible with 3DSt. More importantly, the unique advantage of transparency is that leaflet grasping has been visualized inside the clip arms and its ability to merge anatomic data with 3D color Doppler improved the localization of the residual jet origin. Even though we could not demonstrate that 3DGl may change the strategy and planning of the procedure (beyond the scope of our study) these details obtained only with this new method may facilitate the understanding of suboptimal results and the decision to add a second or third clip.

### Limitations

This study has several limitations. First, it was performed in a single center and data evaluated by experts in the field of 3D echocardiography. Moreover, all image manipulations were performed on stored 3D datasets. Standard 3D, 3DTr and 3DGl methods have slightly different optimal acquisition settings that could not be utilized in this retrospective study. Therefore, it may be that when applied live, 3DTr and 3DGl are even better because dataset can be acquired at their optimal settings rather than generating images from volumes optimized to standard settings.

However, in the absence of a standardization of the method our retrospective analysis demonstrated only a “potential” superiority to the traditional 3D over the new rendering modalities and an online prospective series is advocated to demonstrate the feasibility and usefulness of these new methods throughout the procedure. Moreover, a standardization of the use and positioning of the light source to create the best visualization of the valve clip and catheters may further simplify this new methodology and may reduce the time of manipulation of the images during the procedure. This standardization may also partially reduce the perception of these new tools that remain subjective and may be different between operators.

Therefore, these data may be limited by the lack of comparison of prospective results of MC procedure under the standard 2D-3D TEE monitoring versus the novel 3DTr-3DGl methods. However, this does not detract much from our results since our study design permitted a side-by-side comparison of the different 3DTEE rendering. Further studies are needed to verify our results in different centers and in a larger study population.

## 5. Conclusions

Monitoring and guidance of MC procedure may be improved by this new 3DTr-3DGl rendering and specifically transillumination and transparency facilitate a comprehensive evaluation of all technical steps throughout the procedure. The method is easy, instantaneous (utilizing touch screens) and allow better qualitative and quantitative data of the MV morphology before and after the implantation, better visualization of grasping of the leaflet and recognition of residual MR jets.

## Figures and Tables

**Table 1:** Clinical and procedural characteristics of the study population.

N° of Patients	30
Age (Years)	79 ± 8
Males/Females	14/16
BSA (m^2^)	1.7 ± 0.2
Functional MR (N°)	5
Primary MR (N°)	25
Procedural details	
1 clip implanted (N° of cases)	17
NTR	5
XTR	12
2 Clip implanted (N° of cases)	13
NTR + NTR	1
NTR + XTR	4
XTR + XTR	8

**Table 2:** Quality score comparison (Likert scale from 1, the worst, to 5, the best) among the three different 3DTEE display modalities in the anatomical features definition.

	Standard 3DTEE Display		3DTEE True View		3DTEE True View with Transparency		*p*-Value
	Mean ± SD	Median	Mean ± SD	Median	Mean ± SD	Median	
Pre-procedural mitral valve anatomy	3.5 ± 0.6	4.0	4.0 ± 0.8 *	4.0	4.8 ± 0.5 * †	5.0	<0.001
Post-procedural mitral valve anatomy	3.5 ± 0.6	3.5	3.8 ± 0.6 *	4.0	4.3 ± 0.5 * †	4.0	<0.001
Pre-procedural mitrale regurgitation color jet	3.6 ± 0.6	4.0	3.9 ± 0.6 *	4.0	4.3 ± 0.6 * †	4.0	<0.001
Post-procedural mitral regurgitation color jet	3.5 ± 0.6	4.0	3.8 ± 0.6 *	4.0	4.1 ± 0.7 * †	4.0	<0.001
Clip	3.5 ± 0.7	4.0	3.8 ± 0.4 *	4.0	4.8 ± 0.4 * †	5.0	<0.001

* *p* < 0.05 vs. Standard 3DTEE display; † *p* < 0.05 vs. 3DTEE true view.

## Data Availability

The data presented in this study are available on request from the corresponding author.
